# Effects on osteoclast and osteoblast activities in cultured mouse calvarial bones by synovial fluids from patients with a loose joint prosthesis and from osteoarthritis patients

**DOI:** 10.1186/ar2127

**Published:** 2007-02-22

**Authors:** Martin K Andersson, Pernilla Lundberg, Acke Ohlin, Mark J Perry, Anita Lie, André Stark, Ulf H Lerner

**Affiliations:** 1 Department of Orthopaedic Surgery, Karolinska Hospital, Karolinska Institute, 171 76, Stockholm, Sweden; 2 Department of Oral Cell Biology, Umeå University, Umeå, 901 87, Sweden; 3 Department of Orthopaedics, Malmö University Hospital, Lund University, Lund, 205 02, Sweden; 4 Departments of Anatomy and Clinical Sciences North Bristol, University of Bristol, Bristol, BS2 8EJ, UK

## Abstract

Aseptic loosening of a joint prosthesis is associated with remodelling of bone tissue in the vicinity of the prosthesis. In the present study, we investigated the effects of synovial fluid (SF) from patients with a loose prosthetic component and periprosthetic osteolysis on osteoclast and osteoblast activities *in vitro *and made comparisons with the effects of SF from patients with osteoarthritis (OA). Bone resorption was assessed by the release of calcium 45 (^45^Ca) from cultured calvariae. The mRNA expression in calvarial bones of molecules known to be involved in osteoclast and osteoblast differentiation was assessed using semi-quantitative reverse transcription-polymerase chain reaction (PCR) and real-time PCR. SFs from patients with a loose joint prosthesis and patients with OA, but not SFs from healthy subjects, significantly enhanced ^45^Ca release, effects associated with increased mRNA expression of calcitonin receptor and tartrate-resistant acid phosphatase. The mRNA expression of receptor activator of nuclear factor-kappa-B ligand (*rankl*) and osteoprotegerin (*opg*) was enhanced by SFs from both patient categories. The mRNA expressions of *nfat2 *(nuclear factor of activated T cells 2) and *oscar *(osteoclast-associated receptor) were enhanced only by SFs from patients with OA, whereas the mRNA expressions of *dap12 *(DNAX-activating protein 12) and fcrγ (Fc receptor common gamma subunit) were not affected by either of the two SF types. Bone resorption induced by SFs was inhibited by addition of OPG. Antibodies neutralising interleukin (IL)-1α, IL-1β, soluble IL-6 receptor, IL-17, or tumour necrosis factor-α, when added to individual SFs, only occasionally decreased the bone-resorbing activity. The mRNA expression of alkaline phosphatase and osteocalcin was increased by SFs from patients with OA, whereas only osteocalcin mRNA was increased by SFs from patients with a loose prosthesis. Our findings demonstrate the presence of a factor (or factors) stimulating both osteoclast and osteoblast activities in SFs from patients with a loose joint prosthesis and periprosthetic osteolysis as well as in SFs from patients with OA. SF-induced bone resorption was dependent on activation of the RANKL/RANK/OPG pathway. The bone-resorbing activity could not be attributed solely to any of the known pro-inflammatory cytokines, well known to stimulate bone resorption, or to RANKL or prostaglandin E_2 _in SFs. The data indicate that SFs from patients with a loose prosthesis or with OA stimulate bone resorption and that SFs from patients with OA are more prone to enhance bone formation.

## Introduction

Aseptic loosening of a joint prosthesis is associated with remodelling of bone tissue in the vicinity of the prosthesis. Histopathological and morphometric analyses of bone tissues from patients reoperated on due to aseptic loosening have demonstrated enhanced osteoclast formation and bone resorption as well as new bone formation [1-3]. The relative importance of excessive resorption and/or inadequate new bone formation for the periprosthetic loss of bone is not known. The fact that degradation peptides of type I collagen (N-telopeptide cross-links) and increased levels of deoxypyridinoline and pyridinoline crosslinks can be measured in serum and urine from patients with a loosened total hip arthroplasty indicates that bone resorption is an important part of the pathogenesis of aseptic loosening [4,5]. This view is further supported by the notion that synovial fluid (SF) from patients with a failed hip prosthesis can stimulate bone-resorbing activity of isolated mouse osteoclasts [6] and osteoclast formation in mouse bone marrow cultures [7] and in cultures of human peripheral blood monocytes [8]. The finding that serum levels of osteocalcin are increased in patients with a loosened hip prosthesis [4] is compatible with the morphological observation that suggests an increase in bone turnover [1,3] similar to bone remodelling in postmenopausal osteoporosis. Interestingly, SFs from patients with a loosened hip arthroplasty decrease proliferation of human osteoblasts in contrast to the stimulatory effect by SFs from osteoarthritic patients without any prosthesis [9]. These observations suggest that factors present in SF act on osteoblasts to inhibit proliferation and to enhance differentiation. Such a view is also compatible with the notion that positive bone scans are a common finding in the vicinity of loosened hip prosthesis (MK Andersson, P Lundberg, A Ohlin, MJ Perry, A Lie, A Stark, UH Lerner, unpublished observations).

Much effort has been devoted to studies of the presence of cytokines with bone-resorbing activity in periprosthetic tissues, mainly in pseudosynovial membrane surrounding the prosthesis and in SFs. Thus, interleukin (IL)-1α, IL-1β, IL-6, IL-8, IL-11, tumour necrosis factor-alpha (TNF-α), transforming growth factor-β, and platelet-derived growth factor have been found either in the membranes or in supernatants obtained by culturing of such membranes [10-14]. In an attempt to compare the formation of bone-resorbing activity in different periprosthetic tissues, we incubated pseudosynovial membranes and joint capsules from patients with a loosened hip prosthesis and found (stimulating bone resorption of significantly higher activity in cultured neonatal mouse calvariae in supernatants from joint capsules) that supernatants from joint capsules stimulated bone resorption in cultured mouse calvariae significantly more than supernatants from pseudosynovial membranes [15]. This activity was produced mainly by the inner parts of the capsules containing an abundance of macrophage-phagocytosed wear debris [16]. Based upon these findings, we hypothesised that bone-resorbing activity is produced mainly by macrophages in the capsule and that this activity is released to the SF and then penetrates into the periprosthetic tissues. The presence of several cytokines known to stimulate bone resorption in SFs from patients with a loosened hip prosthesis, including IL-1α, IL-1β, IL-6, IL-8, IL-11, oncostatin M, TNF-α, and macrophage colony-stimulating factor [17-21], supports such a view.

The formation of osteoclasts, as well as the activity of these cells, is controlled by stromal cells/osteoblasts partly via cell-to-cell contact. Receptor activator of nuclear factor-kappa-B ligand (RANKL), a cell membrane-bound protein in the TNF ligand superfamily expressed on stromal cells/osteoblasts, interacts with RANK, a cell surface receptor in the TNF receptor superfamily expressed on preosteoclasts and mature osteoclasts [22-26]. This cell-to-cell contact can be inhibited by osteoprotegerin (OPG), a soluble cytokine in the TNF receptor superfamily which is expressed and released by stromal cells/osteoblasts and which blocks the activation of RANK by RANKL due to its affinity to RANKL. RANKL is also expressed by lymphocytes, which may be an important activator of osteoclastogenesis in inflammatory conditions such as rheumatoid arthritis [27]. Mice rendered null for the *rank *and *rankl *genes become osteopetrotic, whereas opg^-/- ^mice develop osteoporosis [22-26]. Downstream of RANK, activation of the transcription factors nuclear factor-kappa-B, activator protein-1, and nuclear factor of activated T cells 2 (NFAT2) has been found to be important in pathways in osteoclast differentiation [22-26], and NFAT2 has been considered the master regulator of osteoclastogenesis [28]. Recently, it has been shown that activation of ITAM (immunoreceptor tyrosine-based activation motifs) in DNAX-activating protein 12 (DAP12) and Fc receptor common gamma subunit (FcRγ) is also crucial for induction of osteoclast formation and that mice rendered null for both DAP12 and FcRγ are unable to form osteoclasts and therefore become osteopetrotic [28,29]. DAP12 and FcRγ are activated by ligand-recognising immunoglobulin (Ig)-like receptors. Besides the fact that osteoclast-associated receptor (OSCAR) is an important receptor associated with FcRγ [28,29], it is not yet known which Ig-like receptors are important in osteoclast progenitor cells, nor is it known what the ligands for these receptors are.

The aims of the present investigation were (a) to study whether any activity affecting bone resorption and osteoblast function can be detected in SFs from patients with a loosened hip prosthesis and periprosthetic osteolysis and, if so, (b) to compare this activity with that in SFs from osteoarthritic patients without any prosthesis, (c) to analyse whether the bone-resorbing activity was due to any cytokine known to stimulate bone resorption, and (d) to investigate whether bone resorption induced by SF was associated with any changes in the mRNA expressions of *rankl*, *rank*, *opg*, *nfat2*, *fcrγ*, *dap12*, and *oscar*. The results indicate that SFs from patients with a loose prosthesis and periprosthetic osteolysis or with OA stimulate bone resorption by a process dependent on the RANKL/RANK/OPG system and that SFs from patients with OA are more prone to enhance bone formation.

## Materials and methods

### Materials

Synthetic bovine parathyroid hormone [PTH-(1–34)] was obtained from Bachem AG (Bubendorf, Switzerland); recombinant human IL-1α, recombinant human IL-1β, recombinant human IL-6, recombinant human soluble IL-6 receptor, recombinant human IL-17, recombinant human TNF-α, mouse OPG fused to human IgG_1 _Fc (OPG/Fc chimera), antisera neutralising human IL-1α, human IL-1β, human soluble IL-6 receptor, human IL-17, or human TNF-α from R&D Systems Europe Ltd. (Abingdon, Oxfordshire, UK); essentially fatty acid-free albumin from Sigma-Aldrich (St. Louis, MO, USA); α-modification of minimum essential medium (α-MEM) from Flow Laboratories (Irvine, Scotland, UK); [^45^Ca]CaCl_2 _and Thermo Sequenase-TM II DYEnamic ET™ terminator cycle sequencing kit from Amersham Biosciences UK, Ltd., now part of GE Healthcare (Little Chalfont, Buckinghamshire, UK); HotStar Taq polymerase kit and QIAquick PCR purification kit from Qiagen Ltd. (Crawley, West Sussex, UK); culture dishes and multiwell plates from Costar, now part of Corning Life Sciences (Acton, MA, USA); radio-immunoassay (RIA) kit for prostaglandin E_2 _(PGE_2_) from Dupont-New England Nuclear Chemicals, now part of PerkinElmer Life and Analytical Sciences, Inc. (Waltham, MA, USA); enzyme-linked immunosorbent assay (ELISA) kits for RANKL and OPG from Biomedica Medizinprodukte GmbH & Co. KG (Vienna, Austria); TRIzol LS reagent, deoxyribonuclease I (amplification grade), and oligonucleotide primers from Invitrogen Ltd. (Paisley, Scotland, UK) or Applied Biosystems (Warrington, Cheshire, UK); kits for real-time polymerase chain reaction (PCR) analysis of FcRγ, DAP12, and OSCAR mRNA expression, fluorescence-labelled probes (reporter fluorescent dye VIC at the 5' end and quencher fluorescent dye TAMRA at the 3' end), and TaqMan Universal PCR Master Mix from Applied Biosystems; and the 1st Strand cDNA Synthesis Kit and PCR Core Kit from Roche Diagnostics GmbH (Mannheim, Germany). Synthetic salmon calcitonin was generously provided by Novartis International AG (Basel, Switzerland), 1,25(OH)_2_-vitamin D3 (D3) by F. Hoffmann-La Roche Ltd. (Basel, Switzerland), and indomethacin by Merck Sharp & Dohme BV (Haarlem, The Netherlands). D3 and indomethacin were dissolved in ethanol; the final concentration of ethanol never exceeded 0.1% and did not by itself affect calcium 45 (^45^Ca) release in mouse calvariae. All other compounds were dissolved in either phosphate-buffered saline or culture medium.

### SF samples

SF samples were obtained from 25 patients with osteoarthritis (OA) (mean age 71 ± 6 years, mean ± standard error of the mean [SEM]) of the hip or knee joint. The hip patients had radiologically verified advanced OA with osteophytes and complete narrowing of the joint line. OA of the knee joint was verified radiologically. SF also was obtained from 31 patients (mean age 74 ± 9 years) who underwent revision total hip arthroplasty due to aseptic loosening and who had primary surgery because of OA (duration of the prosthesis *in situ *was 8 ± 4 years). These patients had radiological bone loss varying between grades 1 (radiolucent lines around the cup and femur component with clinical signs of loosening but no migration) and 3 (severe osteolysis around the cup in three directions and with widening of the medullar expansion of the upper femur) according to the classification of the ENDO-Klinik Hamburg GmbH (Hamburg, Germany) [30]. Samples of SF from patients having hip surgery were aspirated intraoperatively and before incision of the joint capsule. In six cases, SF was collected from healthy volunteers; the samples were aspirated with a syringe during normal sterile conditions. All samples were centrifuged for 10 minutes at 2,500 rpm to remove cells and other debris and then aliquoted before storage at -70°C. The ethical committees of the authors' institutions approved this study, and the recommendations of the Helsinki Declaration were followed.

### Bone organ culture

Parietal bones from 6- to 7-day-old CsA mice were dissected and cut into either calvarial halves (gene expression experiments) or four pieces (bone resorption experiments) [31]. The bones were preincubated for 18 to 24 hours in α-MEM containing 0.1% albumin, antibiotics, and 1 μmol/l indomethacin. After preincubation, the bones were thoroughly washed and subsequently cultured for different time periods in multiwell culture dishes to which were added 1.0 ml indomethacin-free α-MEM containing 0.1% albumin, with or without SFs or test substances. The bones were incubated in the presence of 5% CO_2 _in air at 37°C.

### Animals

CsA mice from our own inbred colony were used in all experiments. Animal care and experiments were approved and conducted in accordance with accepted standards of humane animal care and use as deemed appropriate by the Animal Care and Use Committee of Umeå University (Umeå, Sweden).

### Measurement of bone resorption

Bone resorption was assessed by analysing the release of ^45^Ca from bones prelabelled *in vivo*. Two- to three-day-old mice were injected with 1.5 μCi ^45^Ca, and the amount of radioactivity in bone and culture media was analysed at the end of the culture period. Release of isotope was expressed as the percentage release of the initial amount of isotope (calculated as the sum of radioactivity in medium and bone after culture) [32]. In some experiments, the data were recalculated and the results expressed as percentage of control, which was set at 100%. This allowed for accumulation of data from several experiments. When time course experiments were performed, the mice were prelabelled with 12.5 μCi ^45^Ca and the kinetics of the release of ^45^Ca was analysed by withdrawal of small amounts of medium at the stated time points.

### Gene expression in mouse calvarial bone

Calvarial bones were dissected from 6- to 7-day-old mice (CsA), divided into halves along the sagittal suture, and preincubated with α-MEM with 0.1% albumin, antibiotics, and 1 μmol/l indomethacin overnight. After the preculture period, calvarial bones were incubated in control medium or medium containing either SFs (10%) or D3 (10 nmol/l) for 48 hours. For semi-quantitative reverse transcription-PCR (RT-PCR), bones were homogenised and the RNA extracted from five bones per treatment group was pooled for subsequent analyses. When quantitative real-time RT-PCR was used, bones were homogenised and RNA was extracted from individual bones and subsequently used for analyses.

### RNA extraction and cDNA synthesis

Total RNA was extracted from calvarial bones with TRIzol LS reagent in accordance with the manufacturer's protocol. The RNA was quantified spectrophotometrically and the integrity of the RNA preparations was examined by agarose gel electrophoresis. Extracted total RNA was treated with deoxyribonuclease I to eliminate genomic DNA according to the instructions supplied by the manufacturer. One microgram of total RNA, after DNase treatment, was reverse-transcribed into single-stranded cDNA with a 1st Strand cDNA Synthesis Kit using random primers (for semi-quantitative RT-PCRs) or oligo-p(dT)15 primers (for quantitative real-time PCRs). After incubation at 25°C for 10 minutes and at 42°C for 60 minutes, the avian myeloblastosis virus reverse transcriptase was denaturated at 99°C for 5 minutes, followed by cooling to +4°C for 5 minutes. The cDNA was kept at -20°C until used for PCR.

### Semi-quantitative RT-PCR

First-strand cDNA mixture was amplified by PCR by means of a PCR Core Kit and PC-960G Gradient Thermal Cycler (Corbett Life Science, Sydney, Australia) or Mastercycler Gradient (Eppendorf, Hamburg, Germany). The PCRs for RANK, RANKL, OPG, calcitonin receptor (CTR), tartrate-resistant acid phosphatase (TRAP), cathepsin K, and glyceraldehyde-3-phosphate dehydrogenase (GAPDH) were performed using 1 μl of template, 0.2 μM of each primer, 2.5 U Taq DNA polymerase, 1× PCR buffer, 0.2 mM dNTPs, and 1.5 mM MgCl_2 _(100 μl total volume) (with the exception of those for CTR, which were performed with 1.25 mM MgCl_2_). The conditions for PCR were denaturing at 94°C for 2 minutes, annealing for 40 seconds at 65°C (RANKL, RANK, OPG), 64°C (CTR), 59°C (cathepsin K), 58°C (TRAP), and 57°C (GAPDH) followed by elongation at 72°C for 90 seconds; in subsequent cycles, denaturing was performed at 94°C for 40 seconds. The PCRs for RANKL, RANK, and OPG were initiated with hot start by means of HotStar Taq polymerase. The PCRs of RANKL, RANK, and OPG were performed with a step-down technology in which the primer annealing temperature was decreased by 5°C every five cycles down to 45°C. The sequences of primers, the GenBank accession numbers, the positions for the 5' and 3' ends of the nucleotides for the predicted PCR products, and the estimated size of the PCR products have previously been given [33,34]. The expression of these factors was compared at the logarithmic phase of the PCR. The PCR products were electrophoretically size-fractionated in 1.5% agarose gel and visualised using ethidium bromide. The identity of the PCR products was confirmed using a QIAquick purification kit and a Thermo Sequenase-TM II DYEnamic ET™ terminator cycle sequencing kit with sequences analysed on an ABI 377 XL DNA Sequencer (Applied Biosystems, Warrington, Cheshire, UK). Control assays included PCRs on RNA samples that were not reversed-transcribed and were always negative, indicating that amplification of genomic DNA did not contribute to the products obtained in the PCRs.

### Quantitative real-time RT-PCR

Quantitative real-time RT-PCR analyses of RANKL, RANK, OPG, TRAP, NFAT2, FcRγ, DAP12, OSCAR, CTR, cathepsin K, alkaline phosphase, osteocalcin, and β-actin mRNA were performed using the TaqMan Universal PCR Master Mix kit, the ABI PRISM 7900 HT Sequence Detections System and software (Applied Biosystems, Foster City, CA, USA), and fluorescence-labelled probes (reporter fluorescent dye VIC at the 5' end and quencher fluorescent dye TAMRA at the 3' end) as described previously [35]. The sequences and concentrations of primers and probes, the GenBank accession numbers, and the numbers for the 5' and 3' ends of the nucleotides for the predicted PCR products for RANKL, RANK, OPG, TRAP, NFAT2, CTR, cathepsin K, alkaline phosphatase, osteocalcin, and β-actin have been given previously [33,34,36]. For FcRγ, DAP12, and OSCAR, commercially available kits were used. The reaction conditions were an initial step of 2 minutes at 50°C and 10 minutes at 95°C for 15 seconds, followed by 40 cycles of denaturation at 95°C for 15 seconds and annealing/extension at 60°C for 1 minute. No amplification was detected in samples in which the RT reaction had been omitted (data not shown). To control for variability in amplification due to differences in starting mRNA concentrations, β-actin was used as an internal standard. The relative expression of target mRNA was computed from the target threshold cycle (Ct) values and β-actin Ct values by means of the standard curve method (User Bulletin #2; Applied Biosystems).

### Analysis of RANKL and OPG protein in SFs

The concentrations of RANKL and OPG protein in SFs were assessed using commercially available ELISA kits for RANKL and OPG in accordance with the protocols of the manufacturer [20]. The sensitivity for the RANKL and OPG assays was 0.1 pmol/l.

### Analysis of PGE_2 _in SFs

The concentration of PGE_2 _in SFs was assessed using a commercially available RIA kit in accordance with the instructions of the manufacturer.

### Statistical analysis

Statistical analysis of multiple treatment groups was performed using one-way analysis of variance with Levene's homogenecity test and Bonferroni, Dunnett's two-sided, or Dunnett's T3 *post hoc *test. Results are expressed as means ± SEMs. SEM is shown when the height of the error bar is larger than the radius of the symbol. All experiments were repeated at least twice with comparable results. The semi-quantitative RT-PCR analyses from one individual experiment were repeated at least once with comparable results.

## Results

### Effects of SFs on bone resorption in mouse calvarial bones

**Figure 1:**
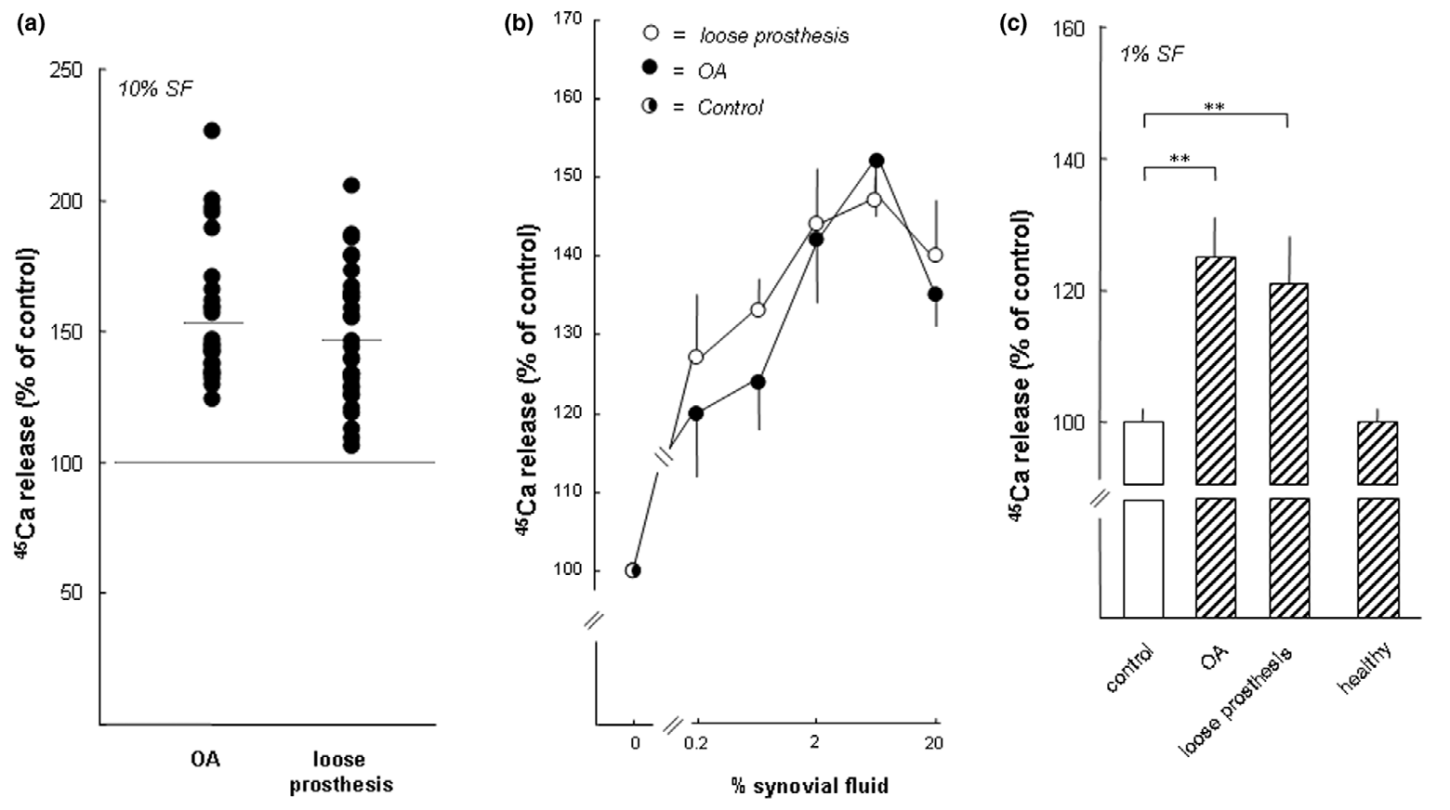
Stimulation of calcium 45 (^45^Ca) release from neonatal mouse calvarial bones by synovial fluids (SFs) from patients with osteoarthritis (OA) and patients with a loose prosthesis, but not by SFs from healthy individuals. **(a) **The effect of SFs from 25 patients with OA and 31 patients with a loose prosthesis. SF from each individual was added to bone culture medium (10%), and each sample was added to five or six bone cultures and incubated for 120 hours. The percentage release of ^45^Ca induced by the different SFs was compared to that observed in unstimulated control bones (100%). Filled circles represent the mean of the effect on ^45^Ca release caused by SF from the individual samples. The effect was statistically different (*p *< 0.05) in 3 of 31 samples from patients with a loose prosthesis and in 25 of 25 samples from patients with OA. **(b) **The concentration-dependent effect on ^45^Ca release by SFs from patients with OA and patients with a loose prosthesis. The data are based on 12 different experiments (SFs from 6 patients with OA and 6 with a loose prosthesis) in which SFs from each patient in each category were incubated as described in **(a) **for 120 hours with five or six calvarial bones and the degree of stimulation was compared to unstimulated bones (100%). Data shown are the cumulative data for six patient samples in each category, and standard error of the mean (SEM) is shown as vertical bars. **(c) **The data from a comparison between SFs (1%) from patients with OA, patients with a loose prosthesis, and healthy subjects. Each sample was incubated for 120 hours with six or seven bones, and ^45^Ca release was compared to unstimulated controls (100%). Values are expressed as mean ± SEM. Asterisks denote statistically significant stimulation (*p *< 0.01).

SF-induced bone resorption was measured by adding SF at various concentrations to mouse bone organ cultures. Initially, SF samples from 25 patients with OA and 31 patients with a loose hip prosthesis were added at a final concentration of 10% to bone culture medium. SFs from 28 of 31 patients with a loose prosthesis were found to cause a statistically significant (*p *< 0.05) stimulation of ^45^Ca release from the calvarial bones (Figure 1a). In 3 of 31 cases, only marginal increase of ^45^Ca release was obtained. Using SF from patients with OA, all samples (25/25) were found to cause a significant (*p *< 0.05) increase in ^45^Ca release. When the data from all patients in the two groups were accumulated, it was found that SFs from the patients with OA caused, on average, a 1.56-fold stimulation of ^45^Ca release and that those from patients with a loose prosthesis a 1.48-fold increase. The average increases of bone resorption observed in the two groups were each statistically significant (*p *< 0.05) compared to untreated control bones but were not statistically different from each other.

Because the potency of the bone-resorbing activity or activities in SF from the two patient groups cannot be reliably assessed by comparing the effects at one concentration, SFs from six patients with OA and six patients with a loose prosthesis were analysed when added to the resorption assay at different concentrations (0.2% to 20%). All 12 samples caused stimulation of ^45^Ca release that was linearly dependent on the concentration of SF (0.2% to 6%) and with a biphasic response at 20%. When the data from all patients in the two groups were accumulated, it was apparent that no difference in the amount of activity stimulating ^45^Ca release could be revealed between patients with OA and patients with a loose prosthesis (Figure 1b).

**Figure 2:**
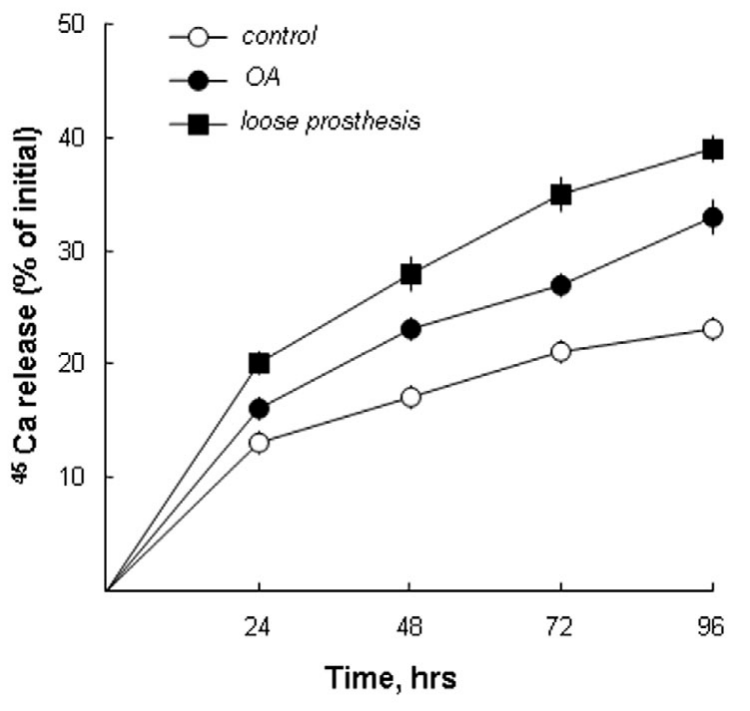
Synovial fluids cause time-dependent stimulation of calcium 45 (^45^Ca) release from neonatal mouse calvarial bones. Synovial fluids from one patient with osteoarthritis (OA) and one patient with a loose prosthesis, at concentrations of 10%, were added to six or seven cultured mouse calvarial bones, and the release of ^45^Ca was compared to that from unstimulated (control) bones. Small amounts of media were withdrawn at the stated time points, and ^45^Ca release was analysed as described in Materials and methods. The data shown represent the absolute percentage of ^45^Ca release. Standard error of the mean is shown as vertical bars when the height of the error bar is larger than the radius of the symbol.

To assess whether the bone-resorbing activity present in SFs from the two patient groups was a unique property of pathological SF, we also obtained SFs from healthy volunteers. Due to the limited amount of fluids that can be obtained from healthy joints, we had to compare the activity at a concentration of 1%. As can be seen in Figure 1c, significant (*p *< 0.01) stimulation of ^45^Ca release was seen when SFs from patients with OA or with a loose prosthesis were added at 1%, which is in agreement with the data in Figure 1b. In contrast, no stimulation of ^45^Ca release from the calvarial bones was obtained with SFs from healthy volunteers at a concentration of 1% (Figure 1c). Stimulation of ^45^Ca release by SFs from patients with OA and patients with a loose prosthesis was dependent on incubation time (Figure 2). Stimulation of ^45^Ca release by SFs (3%) from two out of two patients was significantly inhibited by salmon calcitonin (1 nM; data not shown).

### Effects of SFs on osteoclast differentiation in mouse calvarial bones

**Figure 3:**
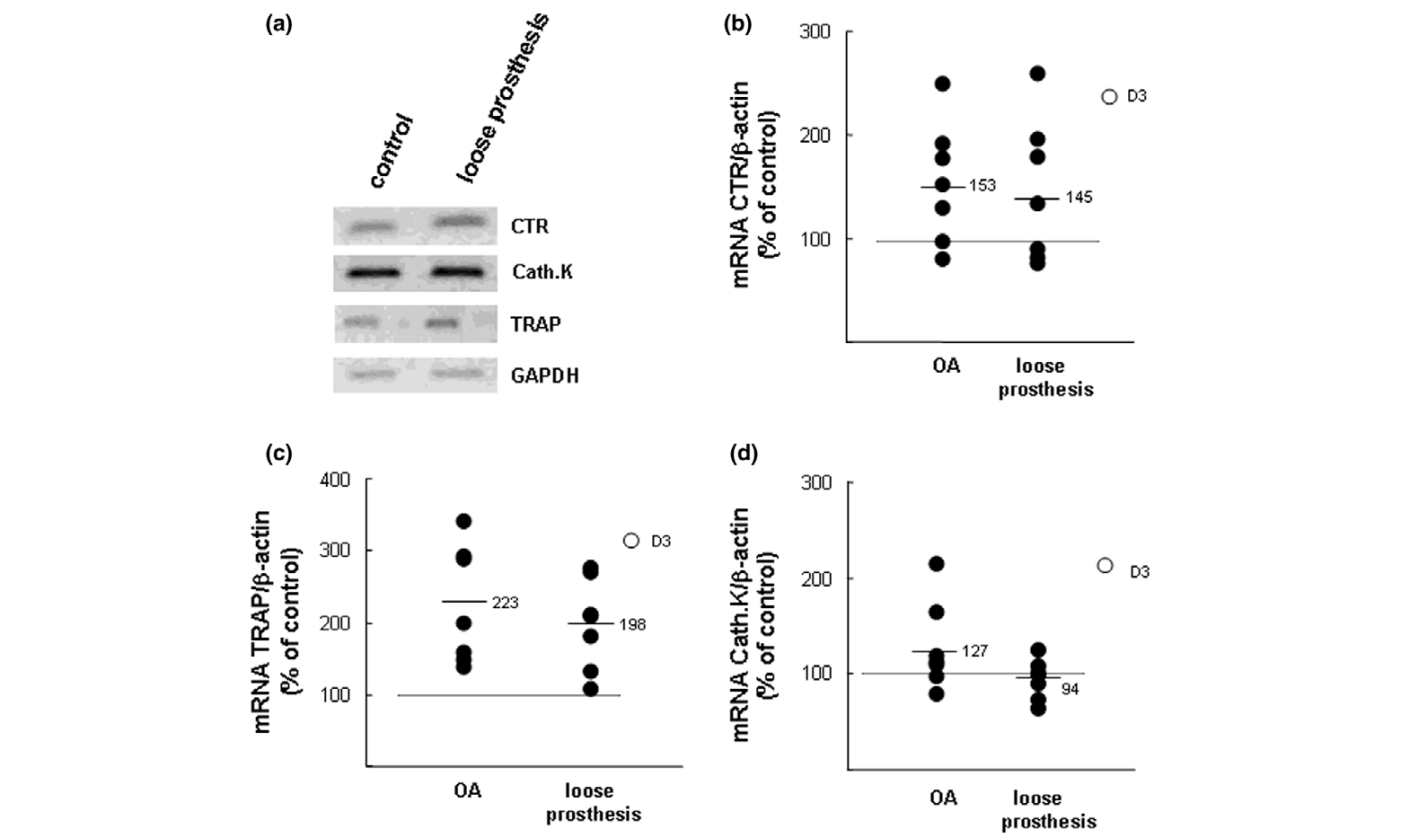
Effects of synovial fluids (SFs) from patients with osteoarthritis (OA) or with a loose prosthesis on the mRNA expressions of the *calcitonin receptor *(CTR), *tartrate-resistant acid phosphatase *(TRAP), and *cathepsin K *(Cath.K) in neonatal mouse calvarial bones. **(a) **Semi-quantitative reverse transcription-polymerase chain reaction (RT-PCR) analysis on samples obtained by incubating five bones in control medium and five in medium containing SF (10%) from a patient with a loose prosthesis. RNA from the five different bones in each group was pooled and used for RT-PCR analysis. **(b) **Quantitative real-time PCR analysis of *calcitonin receptor *(CTR) mRNA in mouse calvarial bones stimulated by SFs from either patients with OA OK or patients with a loose prosthesis. **(c) **Quantitative real-time PCR analysis of *tartrate-resistant acid phosphatase *(TRAP) mRNA in mouse calvarial bones stimulated by SFs from either patients with OA OK or patients with a loose prosthesis. **(d) **Quantitative real-time PCR analysis of *cathepsin K *(Cath.K) mRNA in mouse calvarial bones stimulated by SFs from either patients with OA OK or patients with a loose prosthesis. In **(b-d)**, data were obtained by incubating one calvarial bone for 48 hours with SF (10%) from an OA patient or with SF (10%) from a patient with a loose prosthesis and the effects were compared to those obtained in unstimulated bones (controls = 100%) or in bones stimulated by D3 (10^-8 ^M). Data shown represent the effects by SFs from seven patients with OA and seven with a loose prosthesis. Effects were compared to the means of two unstimulated bones and two bones treated with D3. At the end of the experiments, RNA was extracted and the mRNA expressions were analysed with quantitative real-time PCR. The mRNA expression of the gene of interest was expressed in relation to that of β-actin, used as a housekeeping gene. D3, 1,25(OH)_2_-vitamin D3.

The effect of the SFs on osteoclast differentiation in mouse calvarial bones was assessed by analysing the mRNA expression of three genes known to be upregulated during osteoclastic development. Semi-quantitative RT-PCR showed that SF (10%) from a patient with a loose prosthesis increased the mRNA expression of *ctr *and *trap *but did not cause any change of *cathepsin K *mRNA (Figure 3a). D3 (10 nM) increased the mRNA expression, not only of *ctr *and *trap *but also of *cathepsin K *(data not shown).

To compare the effects of different SFs on osteoclast gene expression, the mRNA expression of the genes encoding *ctr*, *trap*, and *cathepsin K *was also analysed with quantitative real-time PCR by means of seven SFs (10%) from patients with OA and seven SFs (10%) from patients with a loose prosthesis. As appears in Figure 3b, SFs from five of seven patients with OA and four of seven patients with a loose prosthesis enhanced *ctr *mRNA. On average, at a concentration (10^-8 ^M) causing maximal stimulation of bone resorption, the degree of stimulation for the two groups was indistinguishable and slightly less than that caused by D3.

The mRNA expression of *trap *was enhanced by seven of seven SFs from patients with OA and six of seven SFs from patients with a loose prosthesis (Figure 3c). No significant difference between the two groups was found, and the degree of stimulation was slightly decreased compared to that induced by D3 (10^-8 ^M).

Only 2 of 14 SFs stimulated *cathepsin K *mRNA, and those 2 were samples from patients with OA (Figure 3d). In contrast to the SFs, D3 (10^-8 ^M) caused a clear-cut enhanced *cathepsin K *mRNA expression.

### Concentrations of RANKL and OPG in SFs

**Figure 4:**
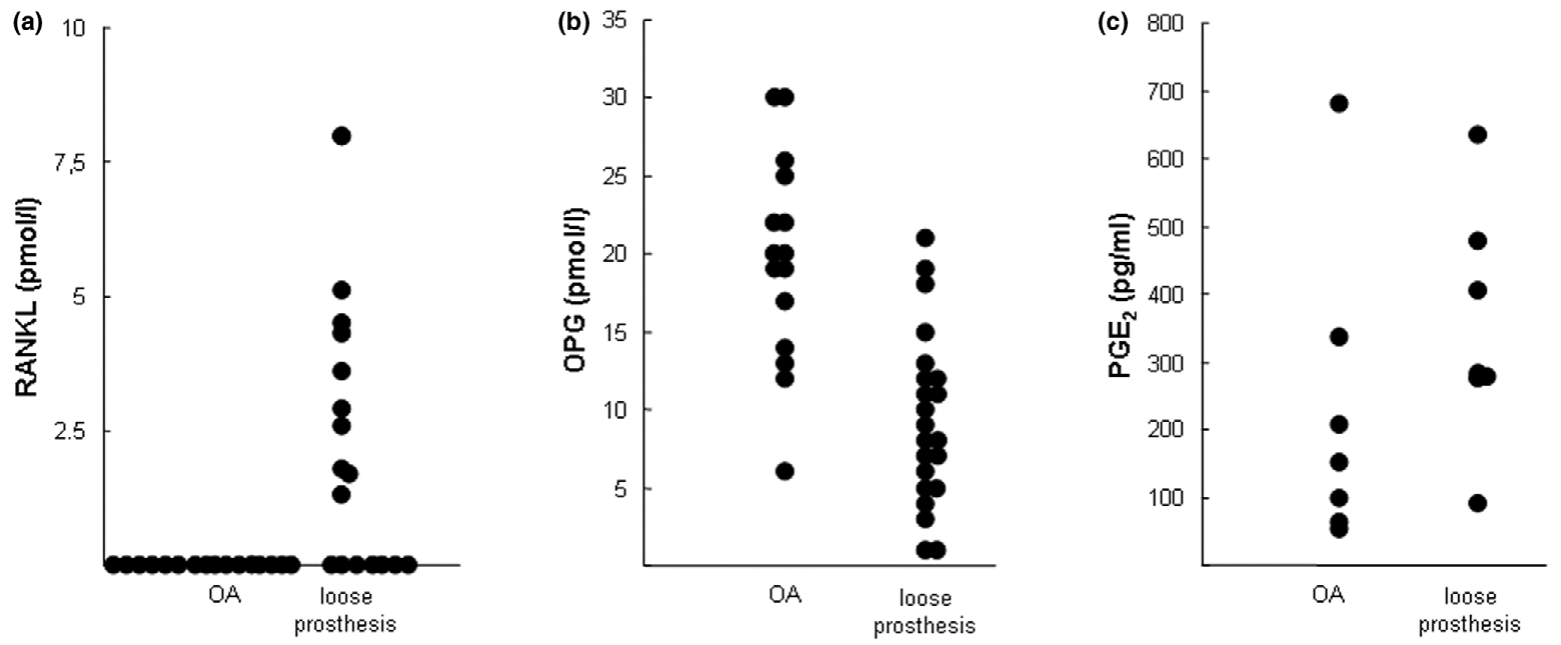
Concentrations of receptor activator of nuclear factor-kappa-B ligand (RANKL), osteoprotegerin (OPG), and prostaglandin E_2 _(PGE_2_) in synovial fluids (SFs) from patients with a loose prosthesis or with osteoarthritis (OA). RANKL **(a) **and OPG **(b) **in SFs were analysed with commercially available enzyme-linked immunosorbent assays. **(c) **PGE_2 _in SFs was analysed with a commercially available radio-immunoassay.

Because RANKL is a potent stimulator of bone resorption, as well as of the expression of *ctr*, *trap*, and *cathepsin K*, in the mouse calvarial system used in the present studies [33,34], we evaluated the possibility that the bone-resorbing activity in the SFs was due to the presence of RANKL by determining the concentrations of RANKL and OPG in the different SFs by means of commercially available ELISAs. As is demonstrated in Figure 4a, RANKL was undetectable (<0.5 pmol/l) in all SFs from patients with OA (*n *= 16) and in 7 of 18 SFs from patients with a loose prosthesis. In 11 of 18 SFs from patients with a loose prosthesis, the concentration of RANKL was 1 to 8 pmol/l (1 to 320 pg/ml) (Figure 4a). The concentrations of OPG were 6 to 30 pmol/l (360 to 1,800 pg/ml) in SFs from patients with OA (*n *= 15) and 1 to 21 pmol/l (60 to 1,260 pg/ml) in SFs from patients with a loose prosthesis (*n *= 22) (Figure 4b). The threshold for action on ^45^Ca release in mouse calvariae by RANKL (in the absence of OPG) is 3 ng/ml, and inhibition by OPG (in the absence of RANKL) is observed at and above 30 ng/ml [34]. Thus, the concentration of RANKL in undiluted SFs (in the resorption assay, the SFs were diluted at least 10 times) is far below that required for stimulation of ^45^Ca release in the mouse calvarial system used.

### Concentration of PGE_2 _in SFs

Prostaglandins have been suggested to be important mediators of bone resorption in the vicinity of loosened joint prosthesis [37]. Because PGE_2 _is the most potent stimulator of bone resorption [37], we evaluated the possibility that the bone-resorbing activity in the SFs was due to PGE_2 _by determining the concentration of PGE_2 _in SF by means of a commercially available RIA. As can be seen in Figure 4c, the concentrations of PGE_2 _varied between 36 and 187 pg/ml in SFs from patients with OA (*n *= 7) and between 46 and 179 pg/ml in SFs from patients with a loose prosthesis (*n *= 7). Because the threshold for action on ^45^Ca release in the mouse calvarial system is 4 ng/ml [38], the concentration of PGE_2 _in the diluted SFs used to stimulate ^45^Ca release was far too low to be responsible for the bone-resorbing activity.

### Expression and importance of RANKL/RANK/OPG in SF-induced bone resorption

**Figure 5:**
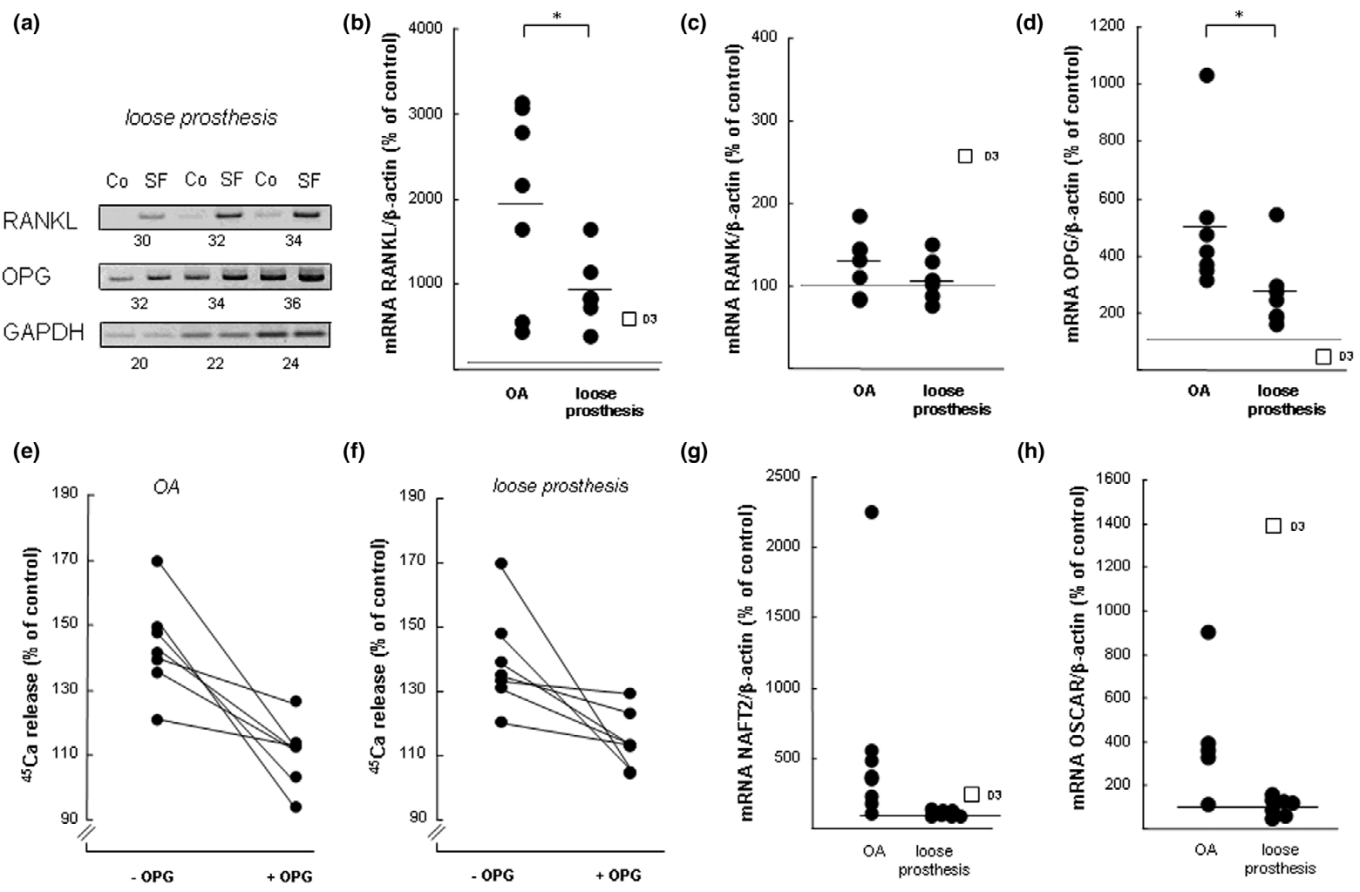
The importance of the RANKL-RANK-OPG pathway in bone resorption induced by synovial fluids (SF) from patients with osteoarthritis (OA) or with a loose prosthesis. **(a) **Semi-quantitative reverse transcription-polymerase chain reaction (RT-PCR) analysis on samples obtained by incubating five bones in control medium and five in medium containing SF (10%) from a patient with a loose prosthesis. RNA from the five different bones in each group was pooled and used for RT-PCR analysis. The expressions of the genes of interest were compared to that of GAPDH, and the values below each gel show the number of cycles in the PCRs. **(b) **Quantitative real-time PCR analysis of *rankl *mRNA in mouse calvarial bones stimulated by SFs from either patients with OA OK or patients with a loose prosthesis. **(c) **Quantitative real-time PCR analysis of *rank *mRNA in mouse calvarial bones stimulated by SFs from either patients with OA or patients with a loose prosthesis. **(d) **Quantitative real-time PCR analysis of *opg *mRNA in mouse calvarial bones stimulated by SFs from either patients with OA OK or patients with a loose prosthesis. In **(b-d)**, one calvarial bone was incubated for 48 hours with SF (10%) from an OA patient or with SF (10%) from a patient with a loose prosthesis and the effects were compared to those obtained in unstimulated bones (controls = 100%) or in bones stimulated by D3 (10^-8 ^M). Data shown represent the effects by SFs from seven patients with OA and seven with a loose prosthesis. Effects were compared to the means of two unstimulated bones and two bones treated with D3. The mRNA expression of the gene of interest was expressed in relation to that of *β-actin*, used as a housekeeping gene. Data shown in **(b-d) **for the different SFs represent the values obtained in individual bones, and the asterisk denotes a statistically significant (*p *< 0.05) effect between averages of SFs from patients with OA and those from patients with a loose prosthesis. **(e) **Addition of OPG to culture medium inhibits calcium 45 (^45^Ca) release induced by SFs from patients with OA. **(f) **Addition of OPG to culture medium inhibits ^45^Ca release induced by SFs from patients with a loose prosthesis. In **(e,f)**, five or six calvarial bones were incubated with SF (10%) from one patient and five or six bones with the same SF and OPG (300 ng/ml). In total, seven patients in each category were tested with and without OPG. At the end of the experiment (96 hours), ^45^Ca release was analysed and compared to that in unstimulated control bones (100%). OPG significantly (*p *< 0.05) inhibited the effect of seven of seven SFs from patients with OA and five of seven OKSFs from patients with a loose prosthesis. **(g) **Quantitative real-time PCR analysis of *nfat2 *mRNA in mouse calvarial bones stimulated by SFs from either patients with OA or patients with a loose prosthesis. **(h) **Quantitative real-time PCR analysis of *oscar *mRNA in mouse calvarial bones stimulated by SFs from either patients with OA or patients with a loose prosthesis. In **(g,h)**, experiments and analysis were performed as described for **(b-d) **above. Co, control; D3, 1,25(OH)_2_-vitamin D3; GAPDH, glyceraldehyde-3-phosphate dehydrogenase; NFAT2, nuclear factor of activated T cells 2; OPG, osteoprotegerin; OSCAR, osteoclast-associated receptor; PCR, polymerase chain reaction; RANK, receptor activator of nuclear factor-kappa-B; RANKL, receptor activator of nuclear factor-kappa-B ligand.

Semi-quantitative RT-PCR assessments of the mRNA expressions of *rankl *and *opg *in mouse calvariae stimulated by SF from a patient with a loose prosthesis indicated that both these molecules were affected. As appears in Figure 5a, bone resorption stimulated by the SF was associated with increased *rankl *and *opg *mRNA. These observations prompted further studies using quantitative real-time PCR and SFs from several patients with OA or with a loose prosthesis.

SFs from all 14 patients, both those with OA and those with a loose prosthesis, caused a robust enhancement of *rankl *mRNA (Figure 5b), which is in agreement with the data obtained by semi-quantitative RT-PCR analysis (Figure 5a). The difference of average stimulation obtained by SFs from patients with OA (20-fold stimulation) from that obtained by SFs from patients with a loose prosthesis (nine-fold stimulation) was statistically significant (*p *< 0.05). The degree of stimulation was slightly larger (SF from patients with a loose prosthesis) and clearly larger (SF from patients with OA) than that caused by D3 (10^-8 ^M; six-fold stimulation). The mRNA expression of *rank *was enhanced by SFs from 6 of 14 patients, but the average effect caused by the two groups of SFs was not different from the control and was clearly less than that obtained by D3 (Figure 5c).

In agreement with the semi-quantitative analysis, *opg *mRNA was enhanced by all 14 SFs (Figure 5d). The average stimulation caused by SFs from patients with OA (five-fold stimulation) was significantly (*p *< 0.05) larger than that caused by SFs from patients with a loose prosthesis (2.7-fold stimulation). In contrast, D3 decreased *opg *mRNA expression, as expected. Stimulation of ^45^Ca release from mouse calvariae by SFs from the two patient groups was significantly (*p *< 0.05) inhibited by OPG in 12 of 14 SFs (Figure 5e,f).

### Expression and importance of NFAT2, OSCAR, FcRγ, and DAP12 in SF-induced bone resorption

The expression of *nfat2 *mRNA in mouse calvarial bones stimulated by SFs from patients with OA was increased in 5 of 6 cases, and the average stimulation was 3.6-fold, which was slightly larger than the 2.2-fold stimulation induced by D3 (Figure 5g). Stimulation of resorption by SFs from patients with a loose prosthesis was not associated with any increase of *nfat2 *mRNA. The difference between SFs from patients with OA and SFs from patients with a loose prosthesis was significantly different (*p *< 0.05). The mRNA expressions of *fcrγ *and *dap12 *were not regulated by SFs from the different patients (data not shown). The mRNA expression of *oscar *was increased by 5 of 6 SFs from patients with OA, with an average 4.2-fold enhancement, whereas SF from patients with a loose prosthesis did not affect *oscar *mRNA (Figure 5h).

### Effects of neutralising antisera on bone-resorbing activity in SFs

The role of different cytokines in SF-induced bone resorption was investigated by incubating mouse bone organ cultures with SFs from patients either with OA or with a loose prosthesis, together with antisera neutralising IL-1α, IL-1β, TNF-α, soluble IL-6 receptor, or IL-17. The specificity and capacity of these antisera were analysed by incubating the antisera with recombinant human IL-1α, IL-1β, TNF-α, and IL-6 in the presence of soluble IL-6 receptor and IL-17. The antisera were found to abolish the stimulatory effect on ^45^Ca release induced by the cytokine assigned to be recognised by the antiserum but were unable to affect stimulation by the other cytokines (data not shown).

**Figure 6:**
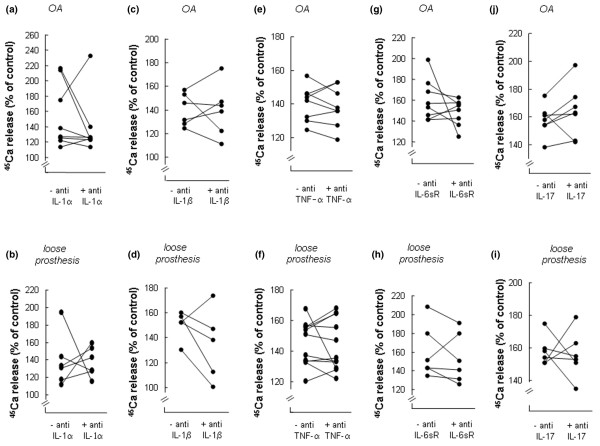
Effects of antisera neutralising different cytokines on calcium 45 (^45^Ca) release from neonatal mouse calvarial bones stimulated by synovial fluids (SFs) from patients with osteoarthritis (OA) or with a loose prosthesis. **(a) **Effect of antiserum neutralising IL-1α on ^45^Ca release induced by SFs from patients with OA. **(b) **Effect of antiserum neutralising IL-1α on ^45^Ca release induced by SFs from patients with a loose prosthesis. **(c) **Effect of antiserum neutralising IL-1β on ^45^Ca release induced by SFs from patients with OA. **(d) **Effect of antiserum neutralising IL-1β on ^45^Ca release induced by SFs from patients with a loose prosthesis. **(e) **Effect of antiserum neutralising TNF-α on ^45^Ca release induced by SFs from patients with OA. **(f) **Effect of antiserum neutralising TNF-α on ^45^Ca release induced by SFs from patients with a loose prosthesis. **(g) **Effect of antiserum neutralising IL-6sR on ^45^Ca release induced by SFs from patients with OA. **(h) **Effect of antiserum neutralising IL-6sR on ^45^Ca release induced by SFs from patients with a loose prosthesis. **(i) **Effect of antiserum neutralising IL-17 on ^45^Ca release induced by SFs from patients with OA. **(j) **Effect of antiserum neutralising IL-17 on ^45^Ca release induced by SFs from patients with a+ loose prosthesis. In each experiment, five or six calvarial bones were incubated for 96 hours with SF (10%) from one patient and five or six bones with the same SF and different antisera. At the end of the experiment (96 hours), ^45^Ca release was analysed and compared to that in unstimulated control bones (100%). IL, interleukin; TNF-α, tumour necrosis factor-alpha.

When antiserum neutralising human IL-1α was added to culture medium containing SFs, it was found that in 3 of 14 patients the stimulation of ^45^Ca release caused by the SFs was inhibited significantly (*p *< 0.05) (Figure 6a,b). For the other SFs, either no or only marginal effect was obtained by anti IL-1α. Similarly, antiserum neutralising human IL-1β caused a significant inhibition (*p *< 0.05) of ^45^Ca release caused by SFs in 3 of 11 patients (Figure 6c,d).

Addition of antiserum neutralising human TNF-α had no effect on the stimulation of ^45^Ca release caused by SFs from 18 of 19 patients. In only one patient, anti-TNF-α significantly (*p *< 0.05) inhibited SF-induced ^45^Ca release (Figure 6e,f).

The bone resorption bioassay used in the present study is not sensitive to human IL-6 unless added together with the soluble IL-6 receptor [34]. To investigate whether stimulation of ^45^Ca release by SFs was dependent on the presence in SFs of both IL-6 and the soluble IL-6 receptor, we added antiserum neutralising human soluble IL-6 receptor. This antiserum significantly (*p *< 0.05) reduced ^45^Ca release induced by 3 of 14 SFs (Figure 6g,h). Antiserum neutralising IL-17 significantly (*p *< 0.05) decreased ^45^Ca release induced by 3 of 13 SFs (Figure 6i,j).

### Effects of SFs on osteoblast differentiation in mouse calvarial bones

By means of quantitative real-time PCR, osteoblastic differentiation was assessed by analysing the mRNA expressions of the enzyme alkaline phosphatase and the bone-specific extracellular matrix protein osteocalcin, both suggested to be associated with mineralisation of bone osteoid and used as clinical parameters of anabolic events in bone.

**Figure 7:**
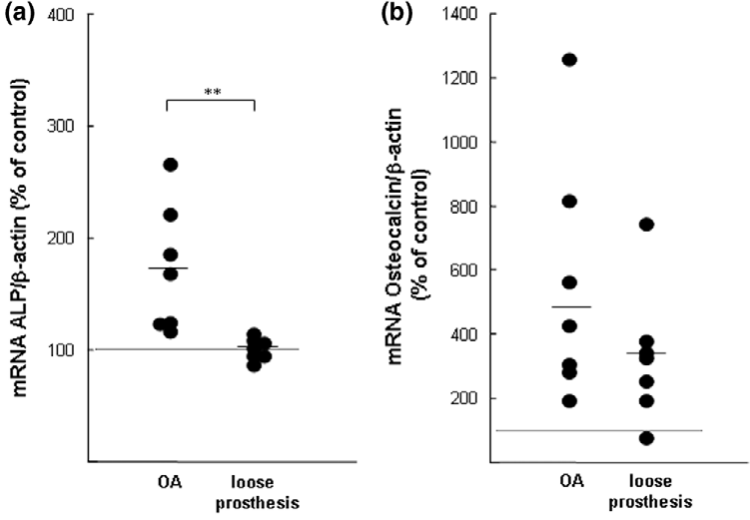
Effects of synovial fluids (SFs) from patients with osteoarthritis (OA) or with a loose prosthesis on the mRNA expressions of *alkaline phosphatase *(ALP) and *osteocalcin *in neonatal mouse calvarial bones. **(a) **Quantitative real-time polymerase chain reaction (PCR) analysis of *alkaline phosphatase *mRNA in mouse calvarial bones stimulated by SFs from either patients with OA or patients with a loose prosthesis. **(b) **Quantitative real-time PCR analysis of *osteocalcin *mRNA in mouse calvarial bones stimulated by SFs from either patients with OA or patients with a loose prosthesis. Calvarial bones were incubated for 48 hours with SFs (10%) from seven OA patients and with SFs (10%) from seven patients with a loose prosthesis, and the effects were compared to those in two unstimulated bones (controls = 100%). At the end of the experiments, RNA was extracted and the mRNA expressions were analysed with quantitative real-time PCR. The mRNA expression of the gene of interest was expressed in relation to that of *β-actin*, used as a housekeeping gene. Data shown for the SFs represent the values obtained by the different SFs in individual bones. Asterisks denote a statistically significant (*p *< 0.01) effect between the averages of SFs from patients with OA and those from patients with a loose prosthesis.

The mRNA expression of alkaline phosphatase was enhanced by 6 of 7 SFs from patients with OA but was unaffected by SFs from patients with a loose prosthesis (Figure 7a). The average difference between the two groups of patients was statistically significant (*p *< 0.01).

SFs from 7 of 7 patients with OA and 6 of 7 patients with a loose prosthesis caused an enhancement of osteocalcin mRNA (Figure 7b). The average degree of stimulation by OA samples was numerically larger than that obtained with samples from patients with a loose prosthesis, but the difference was of borderline significance (*p *= 0.05).

## Discussion

In the present study, we show that SFs from 28 of 31 patients with a loose joint prosthesis and periprosthetic osteolysis significantly stimulate bone resorption in mouse calvariae. In contrast, SF from healthy joints did not cause increased ^45^Ca release. Adding as little as 1% SF was sufficient to cause enhanced resorption. The data provide strong support for the hypothesis that bone-resorbing activity produced in inflammatory processes in periarticular tissues in patients with a loose prosthesis leaks out in the SF, but the data do not provide information on the source of such activity. Based upon our previous observation that bone-resorbing activity, to a large extent, is produced by the synovial capsula [15], we speculate that the bone-resorbing factor or factors present in SF originate mainly from the pseudo-synovial capsules. Our data are in agreement with previous findings showing that SFs from patients with a loose prosthesis can enhance either activation [6] or formation of osteoclasts in cell cultures [7,8] but are the first to show that SFs can stimulate bone resorption in intact bone *ex vivo*. Interestingly, we have recently found that the inflammatory exudate that leaks out into the gingival pocket present in patients with periodontal disease also contains a factor (or factors) stimulating bone resorption in mouse calvarial bones [39,40].

Bone resorption in the mouse calvarial model used in the present study is dependent on initiation of osteoclast formation. Although multinucleated osteoclasts are present in the bones at the time of dissection, these cells disappear during the preculture period and we have reported that subsequent PTH-stimulated resorption is associated with formation of new multinucleated osteoclasts [41]. The finding that the mRNA expression of *ctr *and *trap *was enhanced by the SFs from patients with a loose prosthesis indicates that the bone-resorbing effect is associated with enhanced osteoclastic differentiation. The fact that cathepsin K mRNA was not enhanced does not mean that this enzyme is not important but that osteoclast progenitor cells in the calvarial periosteum are at a late stage and already express several osteoclastic genes.

To get further insight into the mechanisms by which SF stimulated osteoclast differentiation and activity, we analysed the mRNA expressions of *rankl*, *opg*, and *rank*, three molecules of crucial importance for osteoclastogenesis and osteoclast activity [22-26]. It was found that SFs from patients with a loose prosthesis caused an even more robust stimulation of *rankl *mRNA expression than D3 when used at a maximally effective concentration. This was surprising given that the degree of bone resorption caused by the SFs was slightly less than that obtained in bones stimulated by D3. This is most likely explained by the finding that SFs also enhanced *opg *mRNA expression, whereas D3, as expected, decreased *opg *mRNA. In addition, D3 increased *rank *mRNA whereas SFs did not affect the expression of the receptor for RANKL in osteoclast progenitor cells. These findings might help to explain why D3 was a more effective stimulator of bone resorption. The important role of RANKL activation of RANK in SF-induced bone resorption is also indicated by the finding that the stimulatory effect on ^45^Ca release was abolished by the decoy receptor antagonist OPG.

Similar to SFs from patients with a loose joint prosthesis, SFs from patients with OA were found to stimulate bone resorption and osteoclast differentiation. When the bone-resorbing activities of SFs from six patients with OA were compared at a wide range of concentrations with those present in SFs from six patients with a loose prosthesis, we could not observe any quantitative differences. However, *rankl *mRNA was induced to a significantly larger degree by SFs from patients with OA compared to SFs from patients with a loose prosthesis and to an even larger degree than D3. The finding that the two different types of SFs were equipotent as stimulators of bone resorption is most likely explained by the observation that SFs from patients with OA also induced *opg *mRNA to a larger extent than those from patients with a loose prosthesis. These observations indicate that increased RANKL/OPG ratio is an important mechanism causing increased osteoclast differentiation and activity in bone stimulated by OA SFs, a view supported by the finding that OPG inhibited ^45^Ca release caused by SFs from patients with OA. Two previous studies by Kim and colleagues [6,7] reporting stimulation of osteoclast activity and formation by SFs from patients with a loose prosthesis used SFs from patients with OA as controls and did not find any osteoclast-stimulatory effect in the OA samples. In contrast, we used SFs from healthy individuals as negative controls because, as stimulators of bone resorption, SFs from patients with OA were as effective as SFs from patients with a loose prosthesis. We do not know why osteoclasts in intact bones are more sensitive to SFs from OA than osteoclasts in cell cultures. However, our data are in agreement with observations showing that bone resorption is also a feature *in vivo *of patients with OA [42].

The nature of the osteoclast-stimulatory effect present in SFs has not been assessed previously, but the importance of the RANKL/RANK/OPG system was demonstrated in one study showing that OPG could inhibit SF-stimulated osteoclast formation [7]. Cytokines such as IL-1, TNF, IL-6, and IL-17 have all been implicated in the pathogenesis of bone resorption in patients with a loose prosthesis. For several reasons, special attention has been paid to TNF-α: TNF-α can stimulate osteoclast formation from macrophages isolated from pseudomembranes of loosened hip arthroplasties [43], implant particles added to bone marrow macrophages stimulate the production of TNF-α [44], and (even more importantly) polymethymethacrylate particles when implanted in the periosteum of calvariae cause resorption of bone in wild-type animals but not in mice deficient of p55 TNF receptor [44]. In the first attempt to evaluate the importance of these cytokines for the bone-resorptive effect of SFs from patients with a loose prosthesis or with OA, we added antisera specifically neutralising one of these cytokines. Only occasionally did the antisera influence the effects of SFs, suggesting that (in most SFs) the bone-resorbing activity cannot be attributed solely to any of these molecules. However, these observations do not rule out that the cytokines have important roles for the loosening process *in vivo*.

The expressions of the transcription factor NFAT2 and the orphan immunoreceptor OSCAR have been demonstrated to be substantially upregulated in RANKL-stimulated osteoclast progenitor cells, and these molecules have been shown to be crucial for osteoclastogenesis [45,46]. Whether induction of the *nfat2 *and *oscar *genes is important also for periosteal resorption in mouse calvariae is not known. We found that both D3 and SF from patients with OA increased *nfat2 *and *oscar *mRNA, which is in line with the observation that NFAT2 is an important transcription factor in the induction of *oscar *during osteoclastogenesis [47]. However, *oscar *and *nfat2 *mRNA expressions were not affected in bones stimulated by SF from patients with a loose prosthesis. These data indicate that enhanced bone resorption in mouse calvariae is not necessarily dependent on increased NFAT2 and OSCAR, most likely due to the fact that periosteal osteoclast progenitor cells are more highly differentiated than the progenitor cells in hematopoetic tissues. The observations further indicate that the factor or factors responsible for stimulation of bone resorption in mouse calvariae are different in SF from OA patients and that from patients with a loose prosthesis.

Although increased bone resorption is an important pathogenetic mechanism in prosthetic loosening, enhanced bone formation can also be observed in the vicinity of a loose joint prosthesis, and this has been demonstrated by histopathological analysis [1,3], enhanced serum osteocalcin [4], and positive bone scans (MK Andersson, P Lundberg, A Ohlin, MJ Perry, A Lie, A Stark, UH Lerner, unpublished observations). These findings suggest that the inflammatory process in the periarticular tissues in patients with a loose prosthesis causes enhanced bone remodelling, with increased resorption being more dominant than increased bone formation. In the calvarial bones, we observed that SFs from both patients with a loose prosthesis and patients with OA increased the mRNA expression of osteocalcin. The effect of SF from patients with OA was more evident, suggesting a higher degree of anabolic stimulation, which was further indicated by the observation that SFs from patients with OA also enhanced alkaline phosphatase mRNA expression. These observations are in agreement with the clinical experience that patients with OA exhibit increased bone density in the subchondral bone [48] and osteophyte formation and with the fact that the concentrations of osteocalcin and alkaline phosphatase are significantly higher in SF from patients with OA compared to those in SF from patients with a loose prosthesis [18].

In summary, we have found that SFs from patients with a loose joint prosthesis and periprosthetic osteolysis contain activity that can stimulate bone resorption in intact bone, an observation in agreement with previous findings showing that such SFs can stimulate osteoclast activity and formation in cell cultures. For the first time, we demonstrate that SFs from patients with OA also can stimulate bone resorption. The amount of factor (or factors) stimulating osteoclasts seems to be equal in SFs from the two patient categories, but the amount of activity stimulating osteoblast activity seems to be less in SFs from patients with a loose prosthesis. The latter observation, together with our previous finding that SFs from patients with OA contain more insulin-like growth factor-I compared to SFs from patients with a loose prosthesis [49] and increase proliferation of primary human osteoblast-like cells whereas SFs from patients with a loose prosthesis inhibit proliferation of these cells [9], might help to explain why osteoarthritic patients have increased periarticular bone mass and those with a loose prosthesis have decreased bone mass.

## Conclusion

SFs from patients with a loose prosthesis and periprosthetic osteolysis or with OA contained factor (or factors) stimulating bone resorption in mouse calvariae. This effect was associated with increased mRNA expression of molecules reflecting osteoclast differentiation and with increased *rankl *mRNA. The bone-resorbing activity could not be explained solely by the presence of any of the cytokines known to stimulate bone resorption, including RANKL, PGE_2_, IL-1, TNF-α, IL-6, or IL-17. The mRNA expression of alkaline phosphatase and osteocalcin was more highly increased by SFs from patients with OA than those from patients with a loose prosthesis. The data indicate that SFs from patients with a loose prosthesis or with OA stimulate bone resorption and that those from patients with OA are more prone to enhance bone formation.

## Abbreviations

α-MEM = alpha-modification of minimum essential medium; ^45^Ca = calcium 45; Ct = threshold cycle; CTR = calcitonin receptor; D3 = 1,25(OH)_2_-vitamin D3; DAP12 = DNAX-activating protein 12; ELISA = enzyme-linked immunosorbent assay; FcRγ = Fc receptor common gamma subunit; GAPDH = glyceraldehyde-3-phosphate dehydrogenase; Ig = immunoglobulin; IL = interleukin; NFAT2 = nuclear factor of activated T cells 2; OA = osteoarthritis; OPG = osteoprotegerin; OSCAR = osteoclast-associated receptor; PCR = polymerase chain reaction; PGE_2 _= prostaglandin E_2_; PTH = parathyroid hormone; RANK = receptor activator of nuclear factor-kappa-B; RANKL = receptor activator of nuclear factor-kappa-B ligand; RIA = radio-immunoassay; RT-PCR = reverse transcription-polymerase chain reaction; SEM = standard error of the mean; SF = synovial fluid; TNF-α = tumour necrosis factor-alpha; TRAP = tartrate-resistant acid phosphatase.

## Competing interests

The authors declare that they have no competing interests.

## Authors' contributions

MKA participated in study development and collection of patient samples, performed several of the bone organ culture experiments, and actively took part in preparing the draft of the manuscript. PL and AL were responsible for the experiments and analysis of gene expressions. AO was actively involved in the initial work leading to the hypothesis on bone-resorbing activity in SFs, was actively involved in collection of patient samples, and gave substantial input into data evaluation and manuscript preparation. MJP participated in collection of patient samples and gave substantial input into data evaluation and manuscript preparation. AS participated in study development and collection of patient samples and actively took part in preparing the draft of the manuscript. UHL participated in study development and was responsible for the experimental studies and analyses, the evaluation and interpretation of data, and writing the draft of the manuscript. All authors read and approved the final manuscript.
